# Complete Genome Sequences of Mycobacterium farcinogenes Strains Isolated from Clinical Specimens from Patients in Thailand

**DOI:** 10.1128/MRA.01005-21

**Published:** 2021-11-18

**Authors:** Pimpayao Sodsai, Piroon Jenjaroenpun, Nattiya Hirankarn, Thidathip Wongsurawat, Suwatchareeporn Rotcheewaphan

**Affiliations:** a Center of Excellence in Immunology and Immune-Mediated Diseases, Department of Microbiology, Faculty of Medicine, Chulalongkorn University, Bangkok, Thailand; b Division of Immunology, Department of Microbiology, Faculty of Medicine, Chulalongkorn University, Bangkok, Thailand; c Division of Bioinformatics and Data Management for Research, Research Group and Research Network Division, Research Department, Faculty of Medicine Siriraj Hospital, Mahidol University, Bangkok, Thailand; d Division of Bacteriology, Department of Microbiology, Faculty of Medicine, Chulalongkorn University, Bangkok, Thailand; University of Delaware

## Abstract

Mycobacterium farcinogenes is a nonchromogenic, slowly growing mycobacterium that is responsible for bovine farcy. Here, we report two complete genome sequences of Mycobacterium farcinogenes strains isolated from skin and liver abscesses from patients in Thailand.

## ANNOUNCEMENT

Mycobacterium farcinogenes is a nonpigmented, slowly growing mycobacterium that is ubiquitous in the environment. It is a causal agent of bovine farcy, which is a granulomatous inflammation of subcutaneous tissues and lymphatics of cattle, and rarely causes infection in humans. M. farcinogenes is genetically closely related to Mycobacterium senegalense and other members of the Mycobacterium fortuitum group (1, 2). Two isolates of M. farcinogenes were recovered from human liver and facial abscess samples that had been collected at King Chulalongkorn Memorial Hospital (Bangkok, Thailand) in 2019 to 2020. Briefly, clinical specimens were processed using the sodium hydroxide-*N*-acetyl-l-cysteine (NaOH/NALC)-sodium citrate method and incubated on Lowenstein-Jensen medium at 37°C until mycobacterial colonies were visible (3). The colonies were identified as M. fortuitum group with the GenoType Mycobacterium CM v2.0 test (Hain, Germany). Here, Nanopore sequencing was performed for phylogenetic analyses with approval from the institutional review board (IRB) of the Faculty of Medicine, Chulalongkorn University, with exemption from the need for patient consent (certificate of approval 967/2021).

The mycobacteria were subcultured on Lowenstein-Jensen medium at 37°C for 3 to 4 days. Genomic DNA of all samples was extracted using ZymoBIOMICS DNA kits (Zymo Research, USA). DNA purity and DNA concentrations were assessed in all samples with a NanoDrop 2000 spectrophotometer (Thermo Fisher Scientific, USA). In this study, we did not perform DNA shearing or DNA size selection. Library preparation was performed using the rapid barcoding sequencing kit (SQK-RBK004; Oxford Nanopore Technologies [ONT]) prior to sequencing with a flow cell (R10.3/FLO-MIN111; ONT) using a MinION Mk1C system (ONT) for 48 h. The raw signals from the sequencer were base called using a super-high-accuracy model and demultiplexed using Guppy v5.0.7 with a superaccurate DNA model (-c dna_r10.3_450bps_sup.cfg), followed by adapter trimming using Porechop v0.2.4 (https://github.com/rrwick/Porechop). ONT raw reads were filtered using the NanoFilt program (4), and only reads with >500 bases and average quality scores of >10 were stored for *de novo* assembly. The quality of ONT reads was checked with NanoPlot v1.28.1 (https://github.com/wdecoster/NanoPlot). Genome assembly of ONT reads was conducted using Flye v2.8.3 (5), followed by two rounds of polishing with Racon v1.4.10 (−m 8 −x −6 −g −8 −w 500) (6) and a single round of polishing with Medaka v1.4.3 (https://nanoporetech.github.io/medaka). The quality of the draft genomes was examined using QUAST v5.0.2 (7). CheckM v1.1.3 estimated the completeness of CP080510 as 99.78% and that of CP081673 as 99.81%. The GC content of the genomes was calculated using the GC Content Calculator (https://www.sciencebuddies.org/science-fair-projects/references/genomics-g-c-content-calculator). The genomes were classified as M. farcinogenes based on average nucleotide differences and alignment fractions using GTDB-TK v1.5.1 (8) with the Genome Taxonomy Database (GTDB) (9). We used default parameters for all software unless otherwise noted. The genome sequence was annotated by the NCBI Prokaryotic Genome Annotation Pipeline (PGAP) v4.11 (10).

### Data availability.

All circular chromosome sequences and plasmid sequences have been deposited in GenBank under the accession numbers CP080510 and CP081673 to CP081676. The ONT reads are available under BioProject accession number PRJNA749947, which includes accession numbers SRR15372745 and SRR15372744. All genome features are summarized in Table 1.

**TABLE 1:** Genome characteristics of two Mycobacterium farcinogenes strains isolated from clinical specimens in Thailand

GenBank accession no.	Total no. of reads	Read *N*_50_ (bp)	Total sequence length (bp)	GC content (%)	No. of genes	No. of coding sequences	Coding proportion (%)	No. of rRNAs	No. of tRNAs
CP080510	20,720	11,003	6,276,329	66.4	5,976	5,775	96.6	6	48
CP081673	87,975	9,002	6,344,228	66.5	6,055	5,936	98	6	49
CP081674	1,476	8,683	148,235	63.3	145	140	96.6	0	1
CP081675	857	10,799	91,789	63.5	89	85	95.5	0	0
CP081676	989	20,173	23,282	63.8	23	23	100	0	0
