# Erratum for Sodsai et al., “Complete Genome Sequences of Mycobacterium farcinogenes Strains Isolated from Clinical Specimens from Patients in Thailand”

**DOI:** 10.1128/MRA.01145-21

**Published:** 2021-12-09

**Authors:** Pimpayao Sodsai, Piroon Jenjaroenpun, Nattiya Hirankarn, Thidathip Wongsurawat, Suwatchareeporn Rotcheewaphan

**Affiliations:** a Center of Excellence in Immunology and Immune-Mediated Diseases, Department of Microbiology, Faculty of Medicine, Chulalongkorn University, Bangkok, Thailand; b Division of Immunology, Department of Microbiology, Faculty of Medicine, Chulalongkorn University, Bangkok, Thailand; c Division of Bioinformatics and Data Management for Research, Research Group and Research Network Division, Research Department, Faculty of Medicine Siriraj Hospital, Mahidol University, Bangkok, Thailand; d Division of Bacteriology, Department of Microbiology, Faculty of Medicine, Chulalongkorn University, Bangkok, Thailand

## ERRATUM

Volume 10, no. 46, e01005-21, 2021, https://doi.org/10.1128/MRA.01005-21. The article byline should read as given in this correction.

